# Pre-Procedural Vascular Phenotyping Is Associated with Radial Artery Functional Impairment After Transradial Catheterization

**DOI:** 10.3390/jcm15114135

**Published:** 2026-05-27

**Authors:** Xenofon M. Sakellariou, Dimitrios N. Nikas, Panagiotis Papanagiotou, Evangelos Liberopoulos, Eleftheria M. Mastoridou, Antonios Halapas, Theofilos M. Kolettis

**Affiliations:** 1 2nd Department of Cardiology, University Hospital of Ioannina, 45500 Ioannina, Greece; dimitrios.nikas@gmail.com; 2 Department of Radiology, Aretaieion University Hospital, 11528 Athens, Greece; papanagiotou@me.com; 3 1st Propaedeutic Department of Medicine, Laiko Hospital, 11527 Athens, Greece; vaglimp@yahoo.com; 4 1st Department of Internal Medicine, School of Medicine, University of Ioannina, 45221 Ioannina, Greece; e.mastoridou@uoi.gr; 5 Athens Medical Center, 11526 Athens, Greece; ahalapas@gmail.com; 6 Faculty of Medicine, School of Health Sciences, University of Ioannina, 45500 Ioannina, Greece; theofilos.m.kolettis@gmail.com

**Keywords:** transradial access, radial artery, flow-mediated dilation, nitroglycerin-mediated dilation, endothelial function, vascular remodeling, Doppler ultrasound

## Abstract

**Background/Objectives:** Transradial access (TRA) is the preferred route for coronary catheterization, yet its consequences for radial artery vasoreactivity and hemodynamic parameters remain incompletely characterized. We prospectively quantified TRA-induced functional impairment, its clinical determinants, and the association of baseline parameters with post-procedural outcomes. **Methods:** Ninety-four consecutive patients undergoing elective TRA were assessed at baseline, 24 h, and one month using high-resolution Doppler ultrasound. Nine vascular parameters were measured: flow-mediated dilation (FMD), nitroglycerin-mediated dilation (NMD), peak systolic velocity (PSV), resistive index (RI), pulsatility index (PI), resting and hyperemic velocity-time integral, hyperemic blood flow volume, and lumen diameter. Non-parametric methods were applied throughout. **Results:** FMD declined at 24 h (−31.2%; *p* < 0.001) and showed no significant recovery at one month (*p* = 0.08 vs. 24 h). NMD showed a greater acute decline (−36.6%; *p* < 0.001) with partial but statistically significant recovery at one month (*p* < 0.001). PSV recovered fully by one month; RI fell below baseline, consistent with compensatory microvascular vasodilation. Radial artery lumen diameter remained significantly below baseline at one month. Radial artery occlusion occurred in 4 patients (4.3%), all with spontaneous recanalization. Female sex was selectively associated with greater NMD reduction (ΔNMD −8.3% vs. −5.8%; *p* = 0.005) without a statistically significant FMD difference (*p* = 0.40). Older age correlated with impaired FMD recovery at one month (ρ = −0.62; *p* < 0.001) but not with NMD outcomes. Baseline PSV demonstrated the highest discriminatory performance for significant FMD decline (AUC = 0.73). **Conclusions:** TRA causes multidomain, persistent radial artery functional impairment at one month, with distinct recovery trajectories for endothelial and smooth muscle function. Female sex and advanced age are selective determinants of injury and recovery, respectively. A pre-procedural phenotype comprising baseline diameter, PSV, RI, and age is associated with post-procedural outcomes and supports further investigation of pre-procedural phenotyping as a candidate framework for risk stratification.

## 1. Introduction

Transradial access (TRA) has become the preferred choice for coronary catheterization among centers worldwide, with its use increasing substantially over the last decade [1,2,3]. This widespread adoption reflects its well-established advantages over the traditional transfemoral approach, particularly in percutaneous coronary interventions. Maqsood et al. demonstrated a significant reduction in major bleeding and access-site hematoma (OR 0.34; 95% CI 0.24–0.48) [4], while Gargiulo et al. confirmed a mortality benefit (HR 0.77; 95% CI 0.63–0.95; *p* = 0.012) [5], indicating that TRA’s advantages extend beyond bleeding prevention. In chronic total occlusion PCI, fewer access-site complications (OR 0.33) and less major bleeding (OR 0.34) have been reported with similar procedural success [6]. In acute coronary syndrome, particularly STEMI, a mortality benefit (RR 0.71; 95% CI 0.56–0.90) alongside fewer major adverse cardiac events has been observed [7]. These findings have led both the ESC [8,9] and the ACC/AHA/SCAI [10] to recommend radial access as the primary option for coronary catheterization in both acute and chronic coronary syndromes, due to its superior safety profile and clinical outcomes.

Despite these major benefits, TRA is not without consequences for the radial artery itself. The mechanical trauma of arterial puncture, sheath insertion, catheter manipulation, and post-procedural hemostatic compression triggers a cascade of vascular injury that may persist well beyond the peri-procedural period. Optical coherence tomography and high-resolution ultrasonography studies have documented a broad spectrum of structural alterations, ranging from acute intimal tears, reported in 37–67% of patients, to medial dissections occurring in up to 30% of repeat procedures [11,12,13]. Intimal hyperplasia has been observed as early as 2–3 days post-catheterization and shown to persist beyond 30 days [14,15,16], while radial artery lumen diameter has been reported to remain significantly below baseline values at one month and, in some studies, beyond one year [17]. Radial artery occlusion (RAO) represents the most clinically significant complication and, although often silent due to ulnar collateralization, can prevent future use of the vessel for repeat catheterization or as a conduit in coronary artery bypass grafting [18,19,20].

The functional consequences of TRA on the radial artery can be assessed non-invasively through a combination of vasoreactivity testing and Doppler hemodynamic evaluation. Flow-mediated dilation (FMD), the endothelium-dependent vasodilatory response to reactive hyperemia, is a well-validated marker of endothelial function and nitric oxide bioavailability, while nitroglycerin-mediated dilation (NMD) reflects vascular smooth muscle responsiveness independently of endothelial integrity [21,22]. Both parameters can be measured serially using high-resolution Doppler ultrasound without additional patient risk, making them ideal tools for evaluating catheterization-induced vascular injury. Complementary hemodynamic indices, including peak systolic velocity (PSV), resistive index (RI), pulsatility index (PI), and velocity-time integral (VTI), provide additional information on resting and hyperemic flow conditions, downstream vascular resistance, and arterial wall compliance [23,24]. Despite their potential utility, few studies have evaluated both vasoreactivity and Doppler parameters simultaneously across multiple post-procedural timepoints, and the patient-specific factors that predict the severity of functional impairment remain poorly defined.

The radial artery is also clinically important beyond its role as an access site; it is frequently used as a second arterial conduit for coronary artery bypass grafting and is routinely employed for invasive hemodynamic monitoring [25]. Identifying the factors that influence radial artery function and patency after catheterization is therefore a critical focus of current research. We prospectively assessed nine vascular parameters at baseline, 24 h, and one month in 94 consecutive TRA patients, aiming to characterize the temporal course of vascular injury, identify its clinical determinants, and evaluate the predictive value of pre-procedural parameters for post-procedural outcomes.

## 2. Materials and Methods

### 2.1. Study Design and Patients

This prospective, single-center, observational study was conducted at the Department of Cardiology, University Hospital of Ioannina. Patients were recruited sequentially and evaluated at three timepoints: pre-catheterization (baseline), 24 h post-procedure, and one month follow-up. The study was observational and did not alter standard procedural protocols; all clinical decisions remained at the discretion of the treating interventional cardiologist. The study was conducted in accordance with the Declaration of Helsinki and received ethical approval from the Institutional Review Board of the University Hospital of Ioannina prior to enrollment. Written informed consent was obtained from all participants before any study-specific assessment.

Inclusion criteria were: age ≥ 18 years; referral for elective coronary angiography or PCI via TRA; palpable radial pulse at the intended access site; ability to provide written informed consent; and willingness to attend the one-month follow-up visit. Patients were excluded if any of the following were present: non-palpable radial pulse; history of previous ipsilateral transradial catheterization, as prior access can cause persistent endothelial dysfunction and structural remodeling that may confound baseline measurements; known anatomical radial artery anomaly or documented prior RAO; hemodynamic instability requiring emergency intervention; inability to cooperate with ultrasound examination; or acute coronary syndrome requiring urgent catheterization (i.e., STEMI and NSTEMI), as the time constraints of emergency management precluded pre-procedural vascular assessment. Ninety-four patients completed all three assessment timepoints and formed the final study cohort.

### 2.2. Data Collection

A standardized case report form was used to collect all clinical and demographic data at enrollment. Baseline laboratory parameters were obtained from routine pre-procedural blood tests performed within seven days prior to the procedure, including renal function tests, glycated hemoglobin, and eGFR calculated using the CKD-EPI equation. All current medications were recorded in detail at enrollment, including ACE inhibitors, angiotensin II receptor blockers (ARBs), SGLT2 inhibitors, mineralocorticoid receptor antagonists, beta-blockers, diuretics, calcium channel blockers, statins, oral anticoagulants, and antiplatelet agents. All ultrasound measurements and vasoreactivity results were recorded immediately after each assessment and entered into a dedicated electronic database, with all entries reviewed prior to statistical analysis.

### 2.3. Procedural Protocol

All transradial procedures were performed via the right radial artery by experienced interventional cardiologists. Arterial puncture was performed with a palpation-guided 20-gauge needle; after confirming arterial blood return, a 0.025-inch guidewire was inserted and a hydrophilic-coated arterial sheath was positioned using the Seldinger technique. Sheath size was selected based on pre-procedural ultrasound measurement of the arterial diameter, aiming to maintain an outer sheath-to-arterial inner diameter ratio of 1.0 or less whenever possible. In all patients, a 6 Fr introducer sheath (outer diameter 2.52 mm) was used. An intra-arterial antispastic cocktail of verapamil and nitroglycerin was administered immediately after sheath insertion. Unfractionated heparin was given intravenously at a minimum of 50 IU/kg, with additional boluses to maintain an activated clotting time above 200 s during PCI. Patients receiving oral anticoagulation were treated exclusively with direct oral anticoagulants (DOACs); no vitamin K antagonists were used. In accordance with standard peri-procedural practice, the last DOAC dose was administered at least 24 h before the procedure, such that the 24 h post-procedural assessment was performed approximately 48 h after the last dose. Post-procedural hemostasis was achieved using a pneumatic compression device with a patent hemostasis protocol (reverse Barbeau test-guided), targeting 2–4 h of compression duration. Procedures included diagnostic coronary angiography (n = 92), PCI (n = 1), and intravascular ultrasound (n = 1); the predominance of diagnostic procedures reflects the elective nature of the study cohort.

### 2.4. Vascular Ultrasound Assessment

All ultrasound assessments were performed by a single experienced operator using a high-resolution linear vascular transducer (10–15 MHz). Patients were examined supine with the right arm extended in a room maintained at 22–24 °C, following a minimum 10 min rest period to ensure hemodynamic stability. Patients abstained from caffeine, tobacco, and vigorous physical activity for at least 4 h prior to each assessment. The radial artery was imaged 3–4 cm proximal to the styloid process of the radius at the same anatomical site across all timepoints. B-mode imaging was used to measure lumen diameter, followed by pulsed-wave Doppler interrogation with the sample volume centered in the arterial lumen and insonation angle corrected to ≤60°. All measurements were averaged over at least three cardiac cycles. As all measurements were performed by a single experienced operator, inter-observer variability did not apply.

Nine vascular parameters were assessed at each of the three timepoints (baseline, 24 h, and one month): (1) FMD—flow-mediated dilation (%); (2) NMD—nitroglycerin-mediated dilation (%); (3) PSV—resting peak systolic velocity (cm/s); (4) RI—resistive index; (5) PI—pulsatility index; (6) resting VTI—velocity-time integral (cm); (7) hyperemic VTI (cm); (8) hyperemic blood flow volume per beat (mL/beat); and (9) radial artery lumen diameter (mm). Per-beat blood flow volume was calculated as the product of VTI and arterial cross-sectional area derived from the measured lumen diameter.

### 2.5. Vasoreactivity Assessment

FMD was assessed in accordance with the 2019 expert consensus recommendations [26]. A pneumatic cuff was placed on the forearm proximal to the imaging site and inflated to 200 mmHg for five minutes to induce distal ischemia. Upon deflation, reactive hyperemia generated a transient increase in shear stress, stimulating eNOS activity and nitric oxide–mediated endothelium-dependent dilation. The peak shear stimulus was captured at 30–60 s post-deflation, and the peak arterial diameter was recorded at 60–90 s. FMD was calculated as: FMD (%) = (peak hyperemic diameter − baseline diameter)/baseline diameter × 100. The full set of Doppler parameters was simultaneously recorded during the hyperemic phase.

NMD was assessed following a minimum 15 min washout after FMD to allow arterial diameter to return to baseline. Sublingual glyceryl trinitrate 400 μg was administered as an exogenous nitric oxide donor, inducing smooth muscle relaxation independently of endothelial integrity. Continuous ultrasound monitoring was performed for at least five minutes, as peak dilation typically occurs at 3–5 min post-administration. NMD was calculated as: NMD (%) = (peak post-nitroglycerin diameter − baseline diameter)/baseline diameter × 100.

Radial artery patency was assessed at 24 h and one month using pulsed-wave Doppler at the wrist and puncture site. Biphasic or triphasic waveforms confirmed antegrade flow and arterial patency. A monophasic waveform was considered indicative of RAO; color-flow Doppler was used to confirm when waveform interpretation was uncertain. RAO was defined as a complete loss of forward flow. Restoration of antegrade flow at one month in patients with RAO at 24 h was classified as spontaneous recanalization.

### 2.6. Statistical Analysis

All continuous variables were assessed for normality using the Shapiro–Wilk test prior to statistical analysis. Significant departures from normality were identified in all vascular parameters (Shapiro–Wilk *p* < 0.001), necessitating the use of non-parametric methods throughout. The Friedman test was used as a global test for within-subject temporal changes across the three timepoints; significant results were followed by post hoc pairwise comparisons using the Wilcoxon signed-rank test. Between-group comparisons were performed using the Mann–Whitney U test (two groups) or Kruskal–Wallis test (three or more groups). Spearman rank correlation was used to assess associations between continuous variables. ROC curve analysis assessed the discriminative ability of baseline parameters to predict significant post-procedural FMD decline. An available-case analytical strategy was applied, in which each timepoint comparison included all patients with technically valid measurements at the relevant timepoints. The four patients who developed radial artery occlusion were excluded from the 24 h paired comparisons because complete occlusion precludes valid flow-based measurement; following confirmed spontaneous recanalization with restored antegrade flow, valid measurements were again obtainable and these patients were therefore included in the one-month analyses. Exclusion at 24 h was thus determined solely by the technical feasibility of measurement and not by any outcome value. Statistical analyses were performed using IBM SPSS Statistics (version 27, IBM Corp., Armonk, NY, USA).

## 3. Results

### 3.1. Baseline Characteristics

Ninety-four patients were enrolled (69 men [73%]; mean age 65.8 ± 10.6 years). Comorbidities included hypertension (65%), dyslipidemia (78%), type 2 diabetes (30%), smoking history (41%), heart failure (45%), and atrial fibrillation (22%). Mean eGFR was 75.9 ± 15.7 mL/min/1.73 m^2^. Baseline medication use reflected the high cardiovascular risk profile: statins 73%, renin–angiotensin–aldosterone system (RAAS) inhibitors 64%, beta-blockers 48%, antiplatelet agents 45%, diuretics 37% and calcium channel blockers 31%, mineralocorticoid receptor antagonists (MRAs) 27%, oral anticoagulants 21% and sodium-glucose co-transporter 2 inhibitors (SGLT2i) 14%. Mean baseline FMD was 11.58 ± 3.00% and NMD 18.04 ± 4.01%. Resting Doppler parameters confirmed the expected high-resistance pattern of the radial artery at rest: mean PSV 78.62 ± 25.05 cm/s, RI 0.94 ± 0.06, and PI 3.49 ± 0.87. Mean radial artery lumen diameter was 2.43 ± 0.30 mm, approaching the outer diameter of the 6 Fr introducer sheath (2.52 mm), indicating that a substantial proportion of patients had a sheath-to-artery ratio approaching or exceeding unity. Baseline radial artery diameter was significantly smaller in women than in men (2.20 ± 0.25 mm vs. 2.48 ± 0.32 mm; *p* < 0.01). As all patients received a 6 Fr sheath (outer diameter 2.52 mm), this corresponded to a higher sheath-to-artery ratio in women (≈1.15) than in men (≈1.02). All baseline characteristics are detailed in Table 1.

### 3.2. Temporal Changes in Radial Artery Function and Hemodynamic Parameters

#### 3.2.1. Endothelial and Smooth Muscle Vasoreactivity

**Figure 1:**
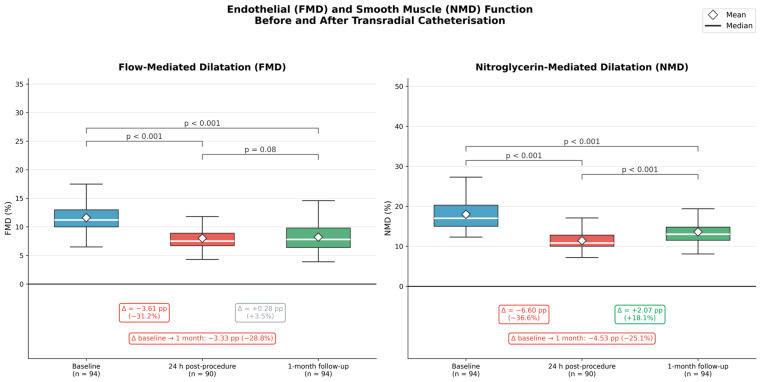
Temporal evolution of FMD and NMD before and after transradial catheterization. Box plots display the distribution of each parameter at baseline (n = 94), 24 h post-procedure (n = 90; four patients excluded due to RAO), and one-month follow-up (n = 94; full sample restored following spontaneous recanalization). Each box represents the interquartile range (25th to 75th percentile), the horizontal white line indicates the median, and the white diamond indicates the mean. Whiskers extend to the most extreme values within 1.5 × IQR of the box edges. The colored annotations below each panel summarize the absolute and relative changes between time points. Both FMD and NMD decreased significantly at 24 h. NMD partially but significantly recovered at one month, whereas FMD showed no significant recovery (*p* = 0.08), suggesting differential recovery trajectories between endothelium-dependent and endothelium-independent vasoreactivity.

FMD declined significantly from 11.58 ± 3.00% at baseline to 7.97 ± 2.04% at 24 h (*p* < 0.001), an absolute reduction of 3.61 percentage points (−31.2% relative decrease). NMD declined from 18.04 ± 4.01% to 11.44 ± 2.52% at 24 h (*p* < 0.001), an absolute reduction of 6.60 percentage points (−36.6% relative decrease). The temporal evolution of FMD and NMD is shown in Figure 1. The magnitude of NMD decline exceeded that of FMD in both absolute and relative terms. Standard deviations of both parameters narrowed at 24 h (FMD: 3.00%→2.04%; NMD: 4.01%→2.52%). At one month (n = 94, full cohort restored following spontaneous recanalization), FMD was 8.25 ± 2.64%—significantly lower than baseline (*p* < 0.001) and not significantly different from the 24 h value (*p* = 0.08)—indicating no significant recovery of endothelial function within the study period. NMD partially recovered to 13.51 ± 2.83% at one month, significantly higher than at 24 h (*p* < 0.001) but remaining significantly below baseline (*p* < 0.001). Inter-individual variability partially re-expanded by one month (FMD SD 2.64%; NMD SD 2.83%). Full results are summarized in Table 2.

#### 3.2.2. Resting Doppler Hemodynamic Parameters

**Figure 2:**
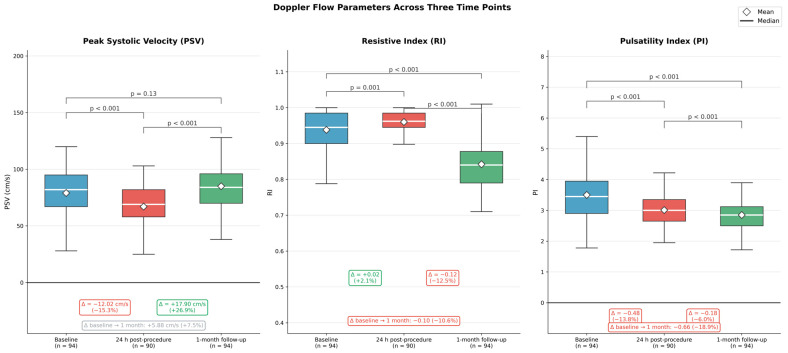
Temporal changes in resting Doppler flow parameters: PSV, RI, PI across the three time points. Each box represents the interquartile range (25th to 75th percentile), the horizontal white line indicates the median, and the white diamond indicates the mean. Whiskers extend to the most extreme values within 1.5 × IQR of the box edges. PSV decreased significantly at 24 h and recovered by one month to values not significantly different from baseline. RI increased modestly at 24 h and decreased significantly below baseline at one month. PI declined progressively across all three time points, with all pairwise comparisons statistically significant.

Resting PSV decreased significantly at 24 h (78.62 ± 25.05 to 66.60 ± 22.10 cm/s; *p* < 0.001) and recovered fully by one month (84.50 ± 18.90 cm/s; *p* = 0.13 vs. baseline). RI increased modestly at 24 h (0.94 ± 0.06 to 0.96 ± 0.03; *p* = 0.001) and fell significantly below baseline at one month (0.84 ± 0.06; *p* < 0.001 vs. both prior time points). PI declined progressively across all three time points (from 3.49 ± 0.87 at baseline to 3.01 ± 0.48 at 24 h and 2.83 ± 0.54 at one month; all pairwise *p* < 0.001). Temporal alterations of PSV, RI, and PI are shown in Figure 2.

#### 3.2.3. Radial Artery Diameter and Hyperemic Flow Reserve

**Figure 3:**
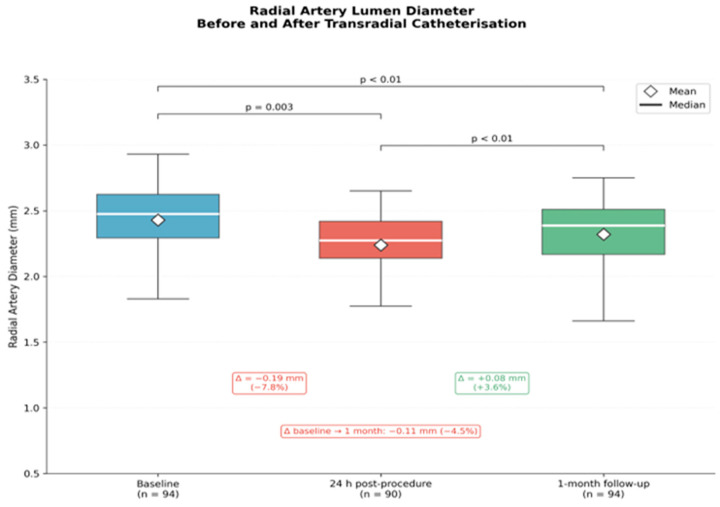
Temporal evolution of radial artery lumen diameter before and after transradial catheterization. Box plots display the distribution of the lumen diameter at baseline (n = 94), 24 h post-procedure (n = 90; four patients excluded due to radial artery occlusion) and one-month follow-up (n = 94; full sample restored following spontaneous recanalization). Each box represents the interquartile range (25th to 75th percentile); the horizontal white line indicates the median and the white diamond indicates the mean. Whiskers extend to the most extreme values within 1.5 × IQR of the box edges. The radial artery lumen diameter decreased significantly at 24 h and recovered partially at one month, remaining however significantly below baseline values.

Radial artery lumen diameter decreased significantly at 24 h (2.43 ± 0.30 to 2.24 ± 0.25 mm; *p* = 0.003; −7.8% relative change) and remained significantly below baseline at one month (2.32 ± 0.28 mm; *p* < 0.01), despite partial recovery compared with the 24 h value (*p* < 0.01) (Figure 3). Hyperemic VTI decreased at 24 h (58.12 ± 16.16 to 55.46 ± 18.45 cm; *p* = 0.01) and rose above baseline at one month (62.81 ± 9.66 cm; *p* = 0.01 vs. baseline; *p* = 0.008 vs. 24 h). Hyperemic blood flow volume decreased at 24 h (2.68 ± 0.98 to 2.19 mL/beat; −18.4%) and returned to near-baseline values at one month (2.66 mL/beat; −0.9% vs. baseline).

### 3.3. Radial Artery Occlusion

Radial artery occlusion was detected at 24 h in 4 patients (4.3%), all male, with a mean age of 62.8 ± 10.6 years. Two distinct clinical profiles were identified: two younger patients (aged 51 and 57 years) had adequate arterial diameters (2.6–3.1 mm; sheath-to-artery ratio <1.0) but markedly impaired baseline vasoreactivity (FMD 6.4–6.9%; NMD 11.1–12.2%); in contrast, two older patients (aged 69 and 74 years) had small radial arteries (both 2.1 mm; sheath-to-artery ratio 1.20) with preserved vasoreactivity. All four patients underwent spontaneous recanalization without intervention, confirmed at one month, and were excluded only from 24 h paired analyses, reducing the effective sample to n = 90 for baseline-to-24 h comparisons.

### 3.4. Subgroup Analysis and Predictors of Functional Damage

#### 3.4.1. FMD Damage by Sex, Diabetes Mellitus, Hypertension, Age and Renal Function

**Figure 4:**
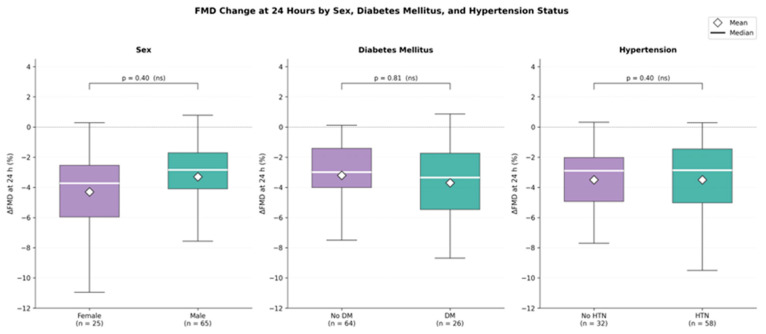
Change in FMD at 24 h stratified by sex (**left**), diabetes mellitus (**center**), and hypertension (**right**). Boxes show median and IQR; diamonds indicate group means. Mann–Whitney U test *p*-values are shown for each comparison.

**Figure 5:**
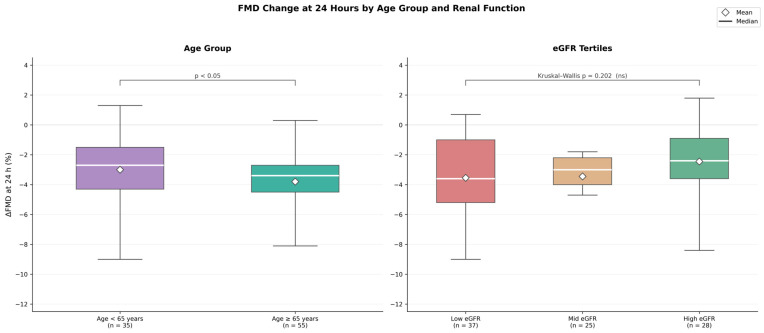
Change in FMD at 24 h stratified by age group (**left**: under 65 versus 65 or older) and eGFR tertile (**right**: low, mid, high). Boxes display median and IQR; diamonds represent group means. Mann–Whitney U test *p*-values are shown for age groups; Kruskal–Wallis *p*-values are shown for eGFR tertiles.

None of the clinical variables tested was significantly associated with the magnitude of FMD reduction at 24 h, with the exception of age (Figure 4). Female patients (n = 25) showed a numerically greater FMD reduction than males (n = 65), with median ΔFMD of approximately −4.3% versus −3.3% (*p* = 0.40). Patients with diabetes mellitus (n = 26) had a median ΔFMD of approximately −3.7% compared with −3.2% in non-diabetic patients (*p* = 0.81). Hypertensive (n = 58) and normotensive patients (n = 32) showed nearly identical reductions (median ΔFMD ≈ −3.5% in both groups; *p* = 0.40). Older patients (≥65 years, n = 55) showed a significantly greater FMD reduction than younger patients (<65 years, n = 35), with median ΔFMD of approximately −3.8% versus −3.0% (*p* < 0.05). Stratification by eGFR tertile revealed no significant differences in ΔFMD at 24 h (Kruskal–Wallis *p* = 0.202) (Figure 5).

#### 3.4.2. NMD Damage by Sex, Diabetes Mellitus, Hypertension, Age, and Renal Function

**Figure 6:**
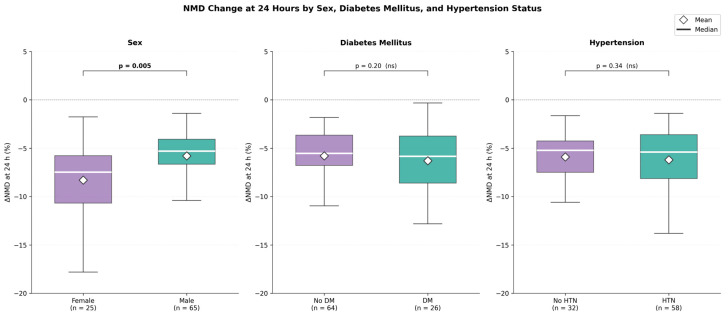
Change in NMD at 24 h stratified by sex (**left**), diabetes mellitus (**center**), and hypertension (**right**). Boxes show median and IQR; diamonds indicate group means. Mann–Whitney U test *p*-values are shown for each comparison.

Female patients experienced significantly greater NMD reduction than males (median ΔNMD −8.3% vs. −5.8%; *p* = 0.005). No significant differences in ΔNMD were observed for diabetes mellitus (*p* = 0.20), hypertension (*p* = 0.34), age group, or eGFR tertile (Figure 6).

#### 3.4.3. Effect of Baseline Medication Use

**Figure 7:**
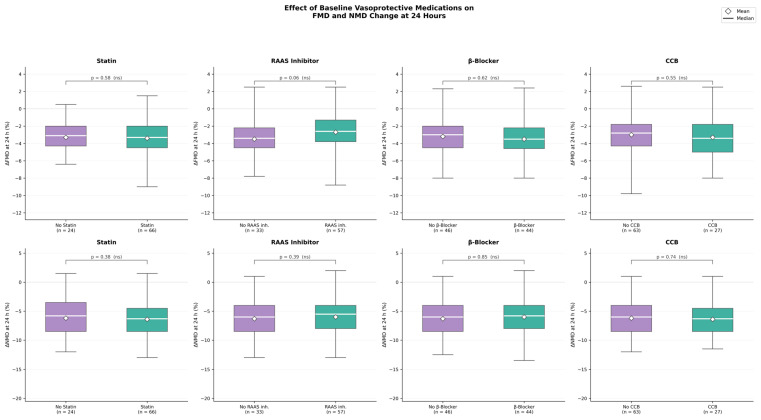
Effect of baseline medication use on ΔFMD% (**upper row**) and ΔNMD% (**lower row**) at 24 h, stratified by statin, RAAS inhibitor, beta-blocker, and CCBs use. Boxes show median and IQR; diamonds indicate group means. Mann–Whitney U test *p*-values are shown for each comparison.

No drug class demonstrated a statistically significant protective effect on post-procedural FMD or NMD at 24 h (Figure 7). Statin therapy showed no significant association with either ΔFMD (*p* = 0.58) or ΔNMD (*p* = 0.38). Beta-blockers and calcium channel blockers showed no significant effects on ΔFMD (*p* = 0.62 and *p* = 0.55, respectively) or ΔNMD (*p* = 0.85 and *p* = 0.74, respectively). RAAS inhibitors approached but did not reach significance for ΔFMD (*p* = 0.06) and showed no significant association for ΔNMD (*p* = 0.39).

### 3.5. Predictive Value of Baseline Vascular Parameters

**Figure 8:**
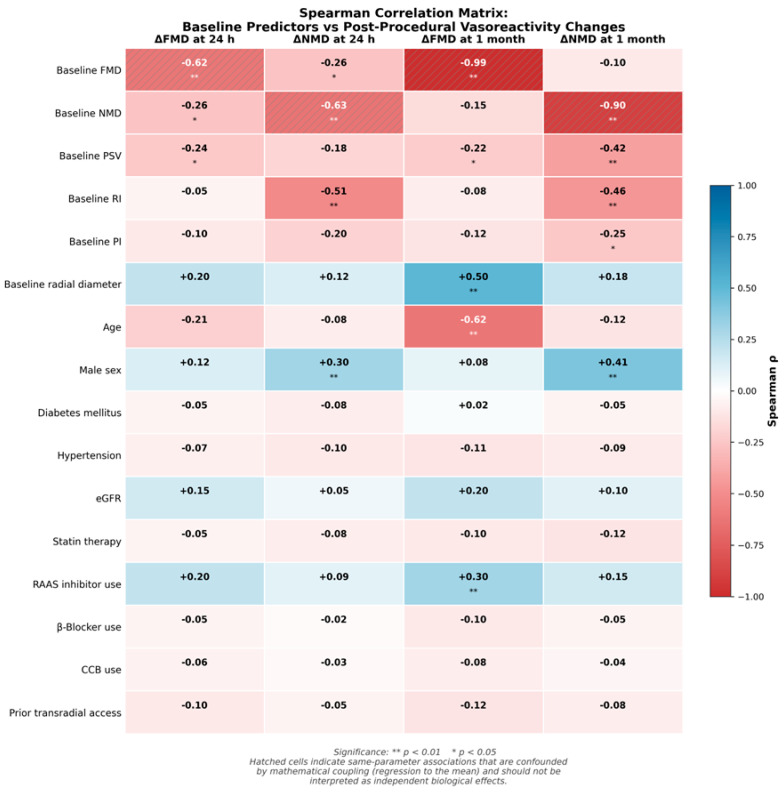
Spearman correlation matrix of baseline clinical, anatomical, and hemodynamic predictors against the four outcome measures: ΔFMD at 24 h, ΔNMD at 24 h, ΔFMD at one month, and ΔNMD at one month. Cell values indicate Spearman rho; significance is indicated by stars (** *p* < 0.01, * *p* < 0.05). Color intensity reflects the magnitude of the correlation (red = negative, blue = positive).

Spearman correlations between baseline vascular parameters and four outcome measures (ΔFMD at 24 h, ΔNMD at 24 h, ΔFMD at one month, and ΔNMD at one month) are presented in Figure 8.

Baseline RI showed a moderate inverse correlation with ΔNMD at 24 h (ρ = −0.51; *p* < 0.001) and at one month (ρ = −0.46; *p* < 0.001). Age was strongly inversely correlated with ΔFMD at one month (ρ = −0.62; *p* < 0.001) but showed only a weak association with ΔFMD at 24 h (ρ = −0.21; *p* = 0.050) and no significant association with ΔNMD at either timepoint. Baseline radial artery diameter showed a moderate positive correlation with ΔFMD at one month (ρ = +0.50; *p* < 0.001). Male sex correlated positively with ΔNMD at 24 h (ρ = +0.30; *p* < 0.01) and one month (ρ = +0.41; *p* < 0.001). Baseline PSV showed inverse correlations with ΔFMD at 24 h (ρ = −0.24; *p* = 0.012), ΔFMD at one month (ρ = −0.22; *p* = 0.04), and ΔNMD at one month (ρ = −0.42; *p* = 0.001).

### 3.6. Discriminatory Performance of Baseline Parameters (ROC Analysis)

**Figure 9:**
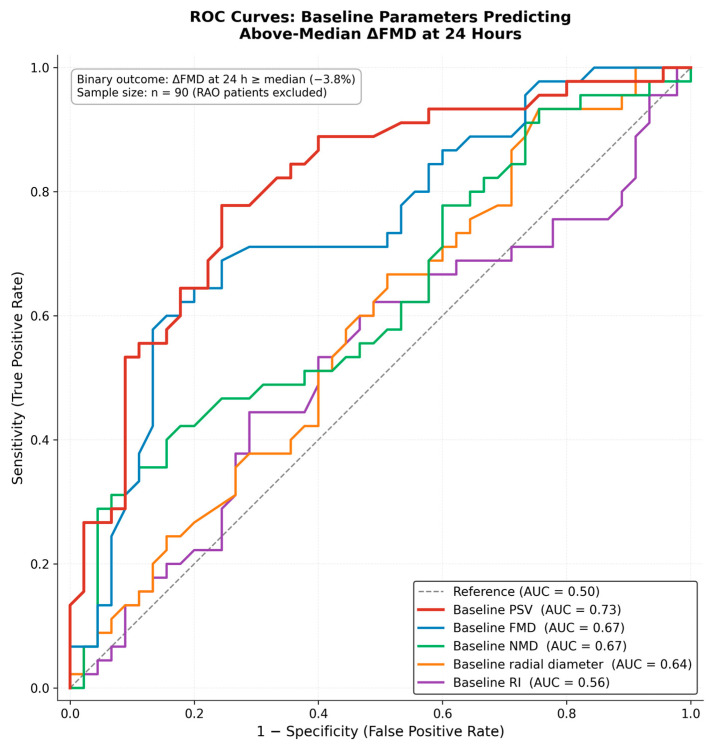
ROC curves for five baseline vascular parameters predicting above-median ΔFMD at 24 h. The binary outcome was defined as a change in FMD at 24 h more negative than the cohort median (ΔFMD ≥ −3.8%). Analyses were performed in the 90 patients with paired baseline and 24 h measurements. Area under the curve (AUC) values are shown in the legend. None of the parameters reached the threshold for excellent discrimination (AUC > 0.80).

The discriminatory ability of five baseline parameters—PSV, FMD, NMD, radial artery diameter, and RI—to identify patients at risk of significant acute FMD decline (defined as ΔFMD at 24 h more negative than the cohort median of −3.8%) was evaluated using ROC curve analysis. Baseline PSV demonstrated the highest discriminatory performance (AUC = 0.73; 95% CI 0.64–0.82). Baseline FMD and NMD each yielded AUC = 0.67 (95% CI 0.56–0.78 and 0.58–0.76, respectively). Baseline radial artery diameter yielded AUC = 0.64 (95% CI 0.54–0.74). Baseline RI showed minimal discriminatory ability (AUC = 0.56; 95% CI 0.44–0.68). None of the five parameters reached the conventional threshold for excellent discrimination (AUC > 0.80). Baseline PSV was strongly inversely correlated with baseline radial artery diameter (ρ = −0.68), indicating that the high discriminatory performance of PSV primarily reflects its inverse relationship with arterial diameter through the continuity equation. The ROC curves are presented in Figure 9.

## 4. Discussion

The present study provides a comprehensive prospective multidomain assessment of radial artery function following TRA cardiac catheterization, simultaneously evaluating nine vascular parameters at three predetermined time points in a cohort of 94 consecutive patients. Three principal findings emerge. First, TRA induces significant, multidomain, and largely persistent functional impairment, with FMD showing no significant recovery between 24 h and one month while NMD shows partial but statistically significant recovery, revealing distinct endothelial and smooth-muscle repair trajectories. Second, two patient characteristics were identified as selective determinants of post-procedural injury: female sex is associated with greater smooth-muscle injury, and older age is associated with impaired endothelial recovery at one month. Third, four baseline parameters—radial artery diameter, resting PSV, resting RI, and patient age—constitute a pre-procedural vascular phenotype that modulates both the magnitude of acute injury and the completeness of subsequent recovery, with baseline PSV showing the highest discriminatory performance in ROC analysis.

The differential recovery of FMD and NMD is mechanistically informative. The greater acute NMD decline (−36.6% vs. −31.2% for FMD) suggests that the tunica media is disproportionately vulnerable to TRA-induced injury, most plausibly through direct circumferential compression of smooth-muscle cells by the introducer sheath, particularly when the sheath-to-artery ratio approaches or exceeds unity. Histopathological studies have documented medial smooth-muscle vacuolization and focal necrosis in sheath-contact arterial segments, supporting this mechanistic interpretation [14]. Endothelial injury, reflected by the reduction in FMD, appears to be mediated primarily by alterations in luminal shear stress and direct endothelial contact with the sheath surface, producing a qualitatively distinct damage pattern from that affecting the tunica media. The more rapid partial recovery of NMD compared with the persistent FMD trajectory is consistent with the greater regenerative capacity of vascular smooth muscle cells relative to the slower, progenitor-cell-dependent process of endothelial repair [27]. The present findings are consistent with previous studies confirming acute FMD impairment at 24 h across different access sites and patient populations [28,29]. While some studies suggest partial FMD recovery by 2–3 months [28,29], the present findings demonstrate that at one month, recovery of endothelial function is not yet detectable.

The fall in RI below baseline at one month, coinciding with the progressive PI decline across all time points, is not paradoxical but biologically coherent. In a muscular conduit artery such as the radial, RI and PI are determined predominantly by downstream microvascular resistance rather than by the wall stiffness of the conduit itself; a progressive reduction in these indices therefore reflects a decrease in distal vascular resistance rather than an increase in conduit stiffness. It represents a compensatory microvascular vasodilation of the hand vascular bed that serves to preserve distal tissue perfusion when proximal conduit artery vasoreactivity remains impaired. This interpretation is internally consistent with the remaining data: the conduit artery shows persistent functional impairment (reduced FMD and NMD) and incomplete structural recovery, with lumen diameter remaining significantly below baseline at one month (2.32 ± 0.28 mm vs. 2.43 ± 0.30 mm; *p* < 0.01), while the distal microcirculation adapts by lowering its resistance. The downstream compensatory mechanism thus operates in parallel with, and is mechanistically distinct from, the impaired vasoreactivity and incomplete structural recovery of the conduit vessel. The complete normalization of PSV by one month, occurring in parallel with persistent FMD and NMD impairment, underscores that restoration of resting hemodynamic adequacy does not reflect recovery of functional vasoreactivity—an important distinction when evaluating radial artery health using Doppler measurements alone.

The selective vulnerability of NMD in women is a novel finding of the present study. Female patients experienced significantly greater NMD reduction than males (median ΔNMD −8.3% vs. −5.8%; *p* = 0.005). By contrast, the corresponding sex difference in FMD reduction was smaller and did not reach statistical significance (*p* = 0.40); the finding is therefore best regarded as a difference in the degree of sex-related vulnerability between the two compartments rather than as an absolute dissociation. Women consistently have smaller radial artery diameters, yielding higher sheath-to-artery ratios and proportionally greater transmural compressive stress on the tunica media [17]. The endothelial layer, comprising a single cell monolayer exposed primarily to luminal shear stress changes, faces comparable perturbations in both sexes when the same external sheath diameter is used, because shear perturbation at the luminal surface is dictated by flow velocity patterns rather than absolute arterial size. The smooth-muscle layer, however, is more directly affected by the transmural compressive stress generated by the sheath, which is inversely related to arterial diameter and thus systematically greater in female patients. The two injury pathways are therefore not entirely independent of vessel size, but differ in their sensitivity to it: compressive injury to the media scales steeply with the sheath-to-artery ratio, whereas endothelial injury carries a substantial size-independent component determined by catheter geometry and flow. Because all patients received an identical 6 Fr sheath, the between-patient variation in injury attributable to vessel size is expected to affect the smooth-muscle compartment more strongly than the endothelial compartment. This framework explains why the sex effect—a proxy for vessel size—is statistically significant for NMD but not for FMD, while remaining compatible with the smaller, non-significant numerical FMD trend observed in women, in whom modestly smaller arteries may also produce somewhat greater, though not statistically distinguishable, endothelial perturbation. These findings support strategies to minimize sheath-to-artery ratio mismatch in women, including pre-procedural ultrasound-guided sheath sizing, smaller sheath selection, sheathless guiding catheter systems, or distal radial access [30,31].

The age-related findings reveal a temporal dissociation in how ageing influences the post-procedural vascular response. The subgroup analysis demonstrated a significantly greater FMD reduction in older patients (≥65 years) at 24 h (*p* < 0.05), though the continuous Spearman correlation was modest (ρ = −0.21; *p* = 0.050), indicating that this age effect at the acute time point is modest and becomes statistically apparent primarily when the cohort is dichotomized. By contrast, age was strongly inversely correlated with ΔFMD at one month (ρ = −0.62; *p* < 0.001), reflecting a substantial age-dependent residual endothelial deficit at this later time point. Critically, age showed no significant association with ΔNMD at either time point, indicating that ageing selectively impairs endothelium-dependent vasoreactivity—both acutely and during the recovery phase—while smooth-muscle function is modulated primarily by mechanical rather than age-related biological factors. This double dissociation—sex acting through mechanical factors (smaller diameter, higher sheath-to-artery ratio) and age acting through biological recovery-capacity factors (reduced endothelial progenitor cell mobilization, impaired nitric oxide bioavailability)—has not previously been reported in the TRA literature.

The pre-procedural vascular phenotype framework identifies four methodologically robust cross-parameter predictors of post-procedural outcome: baseline radial artery diameter, baseline PSV, baseline RI, and patient age. Baseline diameter showed a moderate positive correlation with ΔFMD at one month (ρ = +0.50; *p* < 0.001), consistent with the mechanical-protection hypothesis that larger arteries with lower sheath-to-artery ratios sustain less injury and recover more completely [32,33]. Baseline PSV, which is strongly inversely correlated with radial artery diameter (ρ = −0.68), showed inverse correlations with ΔFMD at 24 h (ρ = −0.24; *p* = 0.012), ΔFMD at one month (ρ = −0.22; *p* = 0.04), and ΔNMD at one month (ρ = −0.42; *p* = 0.001), indicating that higher resting PSV, identifying smaller arteries, is associated with greater post-procedural reductions in vasoreactivity. Baseline RI was inversely correlated with ΔNMD at 24 h (ρ = −0.51; *p* < 0.001) and at 1 month (ρ = −0.46; *p* < 0.001), consistent with greater smooth muscle vulnerability in patients with higher baseline peripheral vascular resistance. Together, these four predictors may constitute a pre-procedural vascular phenotype in which patients with the combined profile of smaller arterial diameter, higher resting PSV, elevated RI, and advanced age represent a potential higher-risk group for substantial acute injury and incomplete recovery.

ROC analysis identified baseline PSV as the parameter with the highest discriminatory performance for predicting significant FMD decline (AUC = 0.73), followed by FMD and NMD (AUC = 0.67 each), radial artery diameter (AUC = 0.64), and RI (AUC = 0.56). None reached the threshold for excellent discrimination (AUC > 0.80). The AUC values for baseline FMD and NMD should be interpreted with caution, as the outcome definition, based on the cohort median ΔFMD, introduces a degree of mathematical coupling with these parameters. By contrast, baseline PSV, diameter, and RI are not subject to this coupling and therefore provide methodologically robust predictive information. The combination of these parameters in a multivariable risk score may yield stronger discriminatory performance than any single parameter alone, representing a priority for future investigation. The 95% confidence intervals for these AUC values are reported in Section 3.6. It should be emphasized, however, that the present analysis is based on cross-parameter correlations and the discrimination of individual baseline parameters; no composite multivariable model, risk score, calibration assessment, internal validation, or external validation was performed. Accordingly, these findings should be regarded as hypothesis-generating, and the development and validation of a formal multivariable prediction model—using appropriate internal and subsequent external validation—is a necessary next step before any clinical prediction tool can be proposed.

The borderline protective signal observed with RAAS inhibitor use, approaching significance in subgroup analysis (*p* = 0.06 for ΔFMD at 24 h) and showing consistent positive Spearman correlations at 24 h (ρ = +0.20) and one month (ρ = +0.30), is biologically plausible given the vasoprotective properties of this drug class through reduction in angiotensin II-mediated oxidative stress, preservation of bradykinin signaling, and upregulation of eNOS activity [34]. However, the high prevalence of RAAS inhibitor use in this cohort (64%), combined with confounding by indication in this observational design, precludes causal inference. A dedicated randomized trial evaluating short-term pre-procedural RAAS inhibition as a vascular-protective strategy before TRA, with ΔFMD at 24 h as the primary endpoint, would be required to test this hypothesis.

A further clinical implication concerns patients in whom the radial artery may subsequently be required as a conduit for coronary artery bypass grafting. The persistence of both endothelial and smooth-muscle impairment at one month—together with incomplete diameter recovery at this time point—suggests that a radial artery harvested within the first month after transradial catheterization may exhibit functional characteristics less favorable than those of a pristine vessel, with potential implications for long-term graft patency. Pre-harvest functional assessment of the radial artery using FMD and NMD may therefore provide valuable complementary information alongside conventional anatomical evaluation in patients who have recently undergone transradial catheterization before bypass surgery.

The present study has several limitations. The single-center design and cohort size of 94 patients limit generalizability and subgroup statistical power; null findings for comorbidities such as diabetes mellitus and hypertension may partly reflect floor effects and insufficient power rather than a true absence of effect. The exclusive use of a 6 Fr sheath precludes assessment of sheath size variation. The absence of a non-catheterized control group introduces the possibility of regression to the mean, though the magnitude, consistency, and mechanistic coherence of the observed changes argue against a purely artifactual explanation. FMD measurement is inherently operator-dependent; adherence to the 2019 consensus guidelines and single-operator assessment mitigated but did not eliminate this limitation. The atrial fibrillation subtype (paroxysmal versus persistent/permanent) was not separately recorded; however, atrial fibrillation is not mechanistically linked to local radial artery vasoreactivity and its status was constant across the within-subject timepoints, making a systematic influence on the principal findings unlikely. In addition, the present study did not develop or validate a formal prediction model: no composite multivariable model, risk score, calibration assessment, internal validation, or external validation was performed, and these represent essential next steps before any clinical prediction tool can be proposed. Finally, the one-month follow-up leaves the long-term trajectory of TRA-induced functional impairment, including whether FMD eventually normalizes or whether inward remodeling progresses-unresolved. Future serial assessments at extended timepoints, such as three, six, and twelve months, are needed to characterize the ultimate course of radial artery functional recovery.

## 5. Conclusions

Transradial cardiac catheterization induces significant, multidomain, and largely persistent functional impairment of the radial artery, extending beyond the immediate post-procedural period to one month. Endothelial function, as assessed by FMD, shows no significant recovery between 24 h and one month within this timeframe, while smooth-muscle vasoreactivity, as assessed by NMD, demonstrates partial but statistically significant recovery—revealing that the two principal vascular compartments follow distinct biological repair pathways following catheter-induced mechanical injury. Resting hemodynamic parameters recover earlier and more completely than vasoreactivity indices, emphasizing that normalization of Doppler flow parameters alone does not reflect restoration of radial artery functional integrity.

Female sex is selectively associated with greater smooth-muscle injury, without a corresponding difference in endothelial function—a novel finding mechanistically attributable to higher sheath-to-artery ratios and greater transmural compressive stress on the tunica media in smaller-diameter vessels. Older age is selectively associated with impaired endothelial recovery at one month rather than with the magnitude of acute injury, highlighting distinct roles of acute susceptibility and regenerative capacity in shaping post-procedural vascular outcomes.

The pre-procedural vascular phenotype, comprising baseline radial artery diameter, baseline PSV, baseline RI, and patient age, emerges as a candidate cross-parameter correlate of the magnitude and persistence of post-procedural functional impairment, warranting formal predictive modeling in future studies. These findings collectively support the integration of structured pre-procedural vascular phenotyping into routine TRA preparation, with particular attention to women, older patients, and those in whom future radial artery integrity is critical for repeat access or surgical bypass grafting. Prospective multicenter studies with extended follow-up are needed to validate this pre-procedural vascular phenotype framework, characterize the long-term natural history of TRA-induced vascular injury, and evaluate targeted pharmacological and technical vascular-protective strategies in high-risk subgroups.

## Figures and Tables

**Table 1:** Baseline patient characteristics (n = 94).

Characteristic	Value
DEMOGRAPHICS	
Age, years (mean ± SD)	65.8 ± 10.6
Male sex, n (%)	69 (73%)
Female sex, n (%)	25 (27%)
CARDIOVASCULAR RISK FACTORS AND COMORBIDITIES	
Hypertension, n (%)	61 (65%)
Dyslipidemia, n (%)	73 (78%)
Diabetes mellitus, n (%)	28 (30%)
Current or prior smoking, n (%)	39 (41%)
Heart failure, n (%)	42 (45%)
Atrial fibrillation, n (%)	21 (22%)
eGFR, mL/min/1.73 m^2^ (mean ± SD; median)	75.9 ± 15.7; 73.5
BASELINE MEDICATION USE	
Statins, n (%)	69 (73%)
RAAS inhibitors, n (%)	60 (64%)
Beta-blockers, n (%)	45 (48%)
Antiplatelet agents, n (%)	42 (45%)
Diuretics, n (%)	35 (37%)
Calcium channel blockers, n (%)	29 (31%)
Mineralocorticoid receptor antagonists, n (%)	25 (27%)
Oral anticoagulants, n (%)	20 (21%)
SGLT2 inhibitors, n (%)	13 (14%)

eGFR = estimated glomerular filtration rate (CKD-EPI equation); RAAS = renin–angiotensin–aldosterone system; SGLT2 = sodium-glucose cotransporter-2; SD = standard deviation.

**Table 2:** Temporal Changes in Radial Artery Vascular Parameters.

Parameter	Baseline (n = 94)	24 h (n = 90) *	1 Month (n = 94)	*p* (Baseline vs. 24 h)	*p* (Baseline vs. 1 Month)	*p* (24 h vs. 1 Month)
VASOREACTIVITY						
FMD (%)	11.58 ± 3.00	7.97 ± 2.04	8.25 ± 2.64	<0.001	<0.001	0.08
NMD (%)	18.04 ± 4.01	11.44 ± 2.52	13.51 ± 2.83	<0.001	<0.001	<0.001
DOPPLER HEMODYNAMICS						
PSV (cm/s)	78.62 ± 25.05	66.60 ± 22.10	84.50 ± 18.90	<0.001	0.13	<0.001
RI	0.94 ± 0.06	0.96 ± 0.03	0.84 ± 0.06	0.001	<0.001	<0.001
PI	3.49 ± 0.87	3.01 ± 0.48	2.83 ± 0.54	<0.001	<0.001	<0.001
Resting VTI (cm)	16.14 ± 4.80	14.00 ± 3.90	18.00 ± 4.40	NS	NS	NS
DIAMETER AND FLOW RESERVE						
Diameter (mm)	2.43 ± 0.30	2.24 ± 0.25	2.32 ± 0.28	0.003	<0.01	<0.01
Hyperemic VTI (cm)	58.12 ± 16.16	55.46 ± 18.45	62.81 ± 9.66	0.01	0.01	0.008
Hyperemic blood flow volume (mL/beat)	2.68 ± 0.98	2.19 ± 0.71	2.66 ± 0.91	<0.001	NS	<0.001

* n = 90 for 24 h analyses (4 patients excluded due to radial artery occlusion). FMD = flow-mediated dilation; NMD = nitroglycerin-mediated dilation; PI = pulsatility index; PSV = peak systolic velocity; RI = resistive index; VTI = velocity-time integral; NS = Non Significant.

## Data Availability

The data presented in this study are available on request from the corresponding author. The data are not publicly available due to privacy.

## Abbreviations

The following abbreviations are used in this manuscript:

ARB	Angiotensin II receptor blocker
AUC	Area under the curve
CCB	Calcium channel blocker
CI	Confidence interval
CKD-EPI	Chronic Kidney Disease Epidemiology Collaboration
DOAC	Direct Oral Anticoagulant
eGFR	Estimated glomerular filtration rate
eNOS	Endothelial nitric oxide synthase
FMD	Flow-mediated dilation
HR	Hazard ratio
MRA	Mineralocorticoid receptor antagonist
NMD	Nitroglycerin-mediated dilation
NO	Nitric oxide
NSTEMI	Non-ST-elevation myocardial infarction
OR	Odds ratio
PCI	Percutaneous coronary intervention
PI	Pulsatility index
PSV	Peak systolic velocity
RAAS	Renin–angiotensin–aldosterone system
RAO	Radial artery occlusion
RI	Resistive index
RR	Relative risk
SD	Standard deviation
SGLT2i	Sodium-glucose cotransporter-2 inhibitor
STEMI	ST-elevation myocardial infarction
TRA	Transradial access
VTI	Velocity-time integral
